# Supplementary material for: Campus healthcare workers’ knowledge and views on injectable pre-exposure prophylaxis prior to rollout in South Africa: A qualitative study
Source: PLOS Glob Public Health. 2026 May 22;6(5):e0006423. doi: 10.1371/journal.pgph.0006423 (PMC13196920; doi:10.1371/journal.pgph.0006423)
Supplement: S1 Appendix — Semi-structured interview guide used to collect qualitative data from participants, including all interview questions and probes. (PDF) [file pgph.0006423.s001.pdf]

Interview Guide: Perceptions and Awareness of Injectable PrEP Among Primary Health Care Workers.

Introduction

Greetings, and Introduction: Hello, my name is Nomzamo Mbatha. Thank you for taking the time to speak with me today.

We are conducting a study to understand the perceptions and awareness of injectable HIV pre-exposure prophylaxis (PrEP) among primary healthcare workers in KwaZulu-Natal, Durban.

Purpose of the Interview:

The purpose of this interview is to gather your thoughts and experiences regarding injectable HIV PrEP.

Your insights will aid us in improving HIV prevention strategies.

Confidentiality Assurance:

Your responses will remain confidential and will only be utilized for research purposes. You may choose not to answer any question or to terminate the interview at any point.

Section 1: Background Information

1. **Demographics and Professional Role**

a) What is your current role in the healthcare system?

b) How long have you been working in primary health care?

c) What type of HIV prevention services do you currently provide?

2. **Experience with HIV Prevention**

a) Have you been involved in providing oral PrEP or other HIV prevention services? If yes, please describe your experience.

b) What are the common challenges you face in delivering HIV prevention services?

Section 2: Awareness and Knowledge of Injectable PrEP

3. **General Awareness**

a) Have you heard about injectable PrEP as a new HIV prevention method? If yes, where did you hear about it?

b) What do you understand about how injectable PrEP works and its benefits?

c) Are you aware of South Africa's recent approval of injectable PrEP?

4. **Knowledge of Effectiveness**

a) In your opinion, how effective do you think injectable PrEP is compared to oral PrEP or other prevention methods?

b) Do you believe injectable PrEP could address challenges with adherence to oral PrEP? Why or why not?

Section 3: Perceived Attributes of Injectable PrEP

5. **Relative Advantage**

a) How do you think injectable PrEP compares to oral PrEP in terms of convenience and effectiveness for patients?

b) What potential advantages do you see for patients in using injectable PrEP?

6. **Compatibility**

a) How do you think injectable PrEP aligns with the current HIV prevention programs at your facility?

b) Do you think patients in your community would be receptive to using injectable PrEP? Why or why not?

7. **Complexity**

a) Do you think injectable PrEP would be easy or difficult to administer and manage in your facility?

b) What training or resources do you think would be necessary to introduce injectable PrEP?

8. **Trialability**

   a) Would you feel comfortable introducing injectable PrEP on a small scale in your clinic before full implementation?

   b) What kind of pilot programs or trials would help you build confidence in this method?

9. **Observability**

   a) What outcomes or feedback would you need to see to be convinced of the value of injectable PrEP?

   b) How would you measure its impact on patients and the community?

Section 4: Barriers and Facilitators

10. **Potential Barriers**

   a) What challenges do you anticipate in implementing injectable PrEP in your facility?

   b) Are there any cultural, logistical, or systemic issues that might hinder its adoption?

11. **Facilitators**

   a) What support would you need from management, policymakers, or other stakeholders to implement injectable PrEP successfully?

   b) How do you think community engagement could support the introduction of injectable PrEP?

Section 5: General Perceptions and Recommendations

12. **Perceptions**

   a) How do you personally feel about the introduction of injectable PrEP in South Africa?

   b) Do you think it has the potential to reduce HIV transmission rates in your community? Why or why not?

13. **Recommendations**

a) What strategies do you think would be most effective for introducing injectable PrEP to healthcare workers and patients?

b) Do you have any additional thoughts or recommendations on how to ensure the success of injectable PrEP in South Africa?
