# Supplementary material for: Campus healthcare workers’ knowledge and views on injectable pre-exposure prophylaxis prior to rollout in South Africa: A qualitative study
Source: PLOS Glob Public Health. 2026 May 22;6(5):e0006423. doi: 10.1371/journal.pgph.0006423 (PMC13196920; doi:10.1371/journal.pgph.0006423)
Supplement: Attachment — Submitted filename: N_N_Mbatha_Response_to_Reviewers_Final 18.03.2026.docx [file pgph.0006423.s003.docx]

**Response to Reviewers**

Manuscript ID: PGPH-D-25-03882

*Campus Healthcare Workers’ Knowledge and Views on Injectable Pre-Exposure Prophylaxis Prior to Rollout in South Africa: A Qualitative Study*

PLOS Global Public Health - Mbatha NN, Mpofana N, Gumede D

**General Response**

We thank the Academic Editor and both reviewers for their thorough and constructive feedback. We have carefully addressed every comment in the revised manuscript. All new or amended text is underlined in the revised manuscript. Page and line numbers below refer to the revised manuscript.

**Editor: Henry Zakumumpa**

| Reviewer comment | Response & action taken | Location (page / line) |
| --- | --- | --- |
| **Comment E1 Academic Editor**  There is consensus from both reviewers around the need to strengthen the discussion section among the areas that need improvement. Please endeavour to make the recommended corrections as much as possible so we can move swiftly to a decision | Thank you. We have substantially revised the Discussion throughout. We reframed the central argument around ‘informed hesitancy’; clarified how findings extend rather than merely review the literature; added a dedicated ‘Implementation Considerations for Campus Settings’ subsection; named study populations for cited studies; and strengthened links between cited evidence and our findings. Full detail is given in the individual reviewer responses below. | **N/A** |
| **Comment R1.1 Reviewer 1**  **Original text: Long-acting injectable PrEP (cabotegravir), administered every two months, has demonstrated superior efficacy compared to daily oral PrEP in multiple clinical trials [7, 8]. In 2022, the WHO recommended its use, and South Africa subsequently approved a phased rollout prioritizing youth-friendly settings [9, 10].**  Cabotegravir is only one example of long acting injectable PrEP and is not available to the SA public beyond clinical trials, while Lenacapavir roll out is planned in South Africa for early 2026. The paragraph makes it sound like Cabotegravir will be rolled out, I suggest discussing the efficacy of Lenacapavir from the Purpose 1 and 2 studies or speak about the efficacy of both products. | We agree the paragraph was misleading. We have revised it to include lenacapavir efficacy data from PURPOSE 1 and PURPOSE 2, and to clarify that South Africa’s approved rollout is of lenacapavir-based injectable PrEP with national implementation planned for 2026.  REVISED TEXT: ‘Long-acting injectable PrEP options include cabotegravir, administered every two months, which demonstrated superior efficacy in the HIV Prevention Trials Network (HPTN) 083 and HPTN 084 clinical trials [7, 8], and lenacapavir, administered every six months, which achieved 100% efficacy among cisgender women in the PURPOSE 1 trial and near-complete protection in the PURPOSE 2 trial among men who have sex with men and transgender individuals. In 2022, the WHO recommended cabotegravir for HIV PrEP, and South Africa has since approved a phased rollout of lenacapavir-based injectable PrEP prioritising youth-friendly settings, with national implementation planned for 2026 [9, 10].’ | **p. 3, lines 63–74** |
| **Comment R1.2 Reviewer 1**  **Original text: All healthcare workers approached agreed to participate in the study; there were no refusals or non-responses.**  Were participants reimbursed? | Thank you. We have added a sentence clarifying that participants were not reimbursed and that interviews were conducted during working hours with the clinic's management's permission.  ADDED TEXT (after p. 6, line 174): ‘Participants were not financially reimbursed or given any token of appreciation for their participation; interviews were conducted during working hours with written permission from campus clinic management.’ | **p. 7, lines 155–160** |
| **Comment R1.3 Reviewer 1**  The discussion is quite lengthy and at times, it is only at surface level, more complexity is required. It would be good to have a bit more succinct summary and focus more on where the results fill in the gaps in literature. | Thank you. We have substantially revised the Discussion: added explicit linking language after each cited study; added a dedicated ‘Implementation Considerations for Campus Settings’ subsection; foregrounded HCW perceptions rather than the literature review; and named populations of all cited studies to make gaps and contributions explicit.  Multiple targeted revisions throughout the Discussion, see individual comment responses below for specific amended passages. | **p. 18–29, lines 385–681 (Discussion)** |
| **Comment R1.4 Reviewer 1**  None of the participants had experience in providing injectable PrEP, it can be expected they do not have knowledge about it, and this is not really adding to any gaps in knowledge in the HIV knowledge base. | Thank you. We agree and have added a dedicated Limitations subsection explicitly acknowledging this. We have also moderated overstated contribution claims in the Discussion.  ADDED new ‘Limitations’ subsection (inserted before Conclusions): ‘A key contextual limitation is that no participant had direct experience administering injectable PrEP, as it was not yet available in South African campus clinics at the time of this study. Findings, therefore, reflect anticipatory rather than experiential knowledge, providing a useful baseline for rollout planning but not capturing the challenges and adaptations that emerge during actual service delivery. Future research should revisit comparable campus clinic settings post-implementation to assess how pre-implementation knowledge, concerns, and optimism evolve in practice. Additionally, purposive sampling from a single university in KwaZulu-Natal limits transferability to other institutional or provincial contexts.’ | **Discussion: Limitations (absent from original)** |
| **Comment R1.5 Reviewer 1**  Campus health clinics face unique constraints, fluctuating student demand driven by academic calendars, heightened privacy concerns within close-knit campus communities, and resource limitations specific to educational institutions. I would have liked to see more mention of these points in both the results and discussion. | Thank you. We have added a dedicated ‘Implementation Considerations for Campus Settings’ subsection addressing these constraints and their implications for injectable PrEP rollout, including the pharmacokinetic risk of missed appointments during long university holidays.  ADDED new subsection ‘Implementation Considerations for Campus Settings’ (inserted in Discussion after p. 25, line 774): addressing academic calendar risks, pharmacokinetic tail risk, seroconversion and resistance risk, mitigation strategies (bridging oral PrEP, aligned scheduling, re-testing protocols), workload burden on campus clinic staff, cold-chain and counselling demands, and relevance to forthcoming lenacapavir rollout. | **p. 4, lines 94–99 (Background); p. 29, lines 663–681 (Discussion)** |
| **Comment R1.6 Reviewer 1**  **Original text: ...lifestyle patterns [5, 6].**  Why would long acting PrEP be better specifically in the student population? | Thank you. We have expanded the background explanation of adherence barriers to specify the behaviours and circumstances that make injectable PrEP particularly advantageous for university students.  REVISED TEXT (p. 2, lines 64–65): ‘...lifestyle patterns such as irregular routines, frequent travel between home and campus, and socially dynamic environments that make discreet, consistent daily pill-taking difficult to sustain [5, 6].’ | **p. 3, lines 55–62 (Background); p. 21, lines 472–487 (Discussion)** |
| **Comment R1.7 Reviewer 1**  One of the worries around implementing long acting prep in tertiary institutions is the long student holidays and not returning for scheduled appointments. As long acting injectables have a long tail period and risk resistance to ARVs if seroconversion occurs, how will these risks be mitigated? | Fully addressed in the new ‘Implementation Considerations for Campus Settings’ subsection (see R1.5). The subsection explicitly names the pharmacokinetic tail risk, seroconversion risk, and resistance risk, and provides specific mitigation strategies.  ADDED new ‘Implementation Considerations for Campus Settings’ subsection addressing these risks. The subsection explicitly names the pharmacokinetic tail risk associated with long-acting injectables: if a student misses a scheduled injection during a university holiday, the prolonged drug-release phase means subtherapeutic drug levels may persist for weeks, creating a window of vulnerability. Should seroconversion occur during this period, exposure to subtherapeutic cabotegravir or lenacapavir concentrations risks selecting for resistance mutations. Mitigation strategies discussed include bridging oral PrEP across holiday periods, scheduling injections to align with the academic calendar, enhanced re-engagement and re-testing protocols at semester commencement, and proactive appointment reminders. These implementation risks make robust pre-rollout planning and student-centred scheduling essential.. | **New content-Discussion** |
| **Comment R1.8 Reviewer 1**  Anticipated improvements are mentioned but what are the barriers or disadvantages? Many HCWs are already overwhelmed and can find new treatment regimens a burden, particularly when it is more medicalized than oral PrEP (and require more choice counselling and time when already overworked). | Thank you. Addressed in the new ‘Implementation Considerations for Campus Settings’ subsection. Workload burden, medicalisation, cold-chain requirements, and intensified counselling demands are now explicitly discussed.  ADDED new ‘Implementation Considerations for Campus Settings’ subsection explicitly discussing barriers and disadvantages for HCWs. Injectable PrEP introduces a more medicalised service model than oral PrEP: each administration requires a trained clinician, a sterile environment, and cold-chain storage, adding logistical demands to already resource-constrained campus clinics. Campus clinic staff are predominantly PHC nurses managing broad primary care caseloads; incorporating injectable PrEP means additional appointment slots, extended counselling sessions covering injection technique, side-effect management (particularly post-injection systemic reactions), and pharmacokinetic risk counselling, all of which compound existing workload pressures. Cold-chain requirements (lenacapavir requires refrigeration) add supply-chain complexity that community-based oral PrEP distribution does not. These barriers require deliberate workforce planning and resource allocation before rollout to avoid placing injectable PrEP implementation on an already overstretched campus clinic workforce. | **New content-Discussion** |
| **Comment R2.1 Reviewer 2**  **Original text: While participants anticipated improvements in HIV prevention through injectable PrEP regarding adherence and reducing stigma...**  “improvements in HIV prevention coverage” | Thank you. the phrase has been made more specific to reflect what participants actually described.  REVISED TEXT (p. 2, line 39): ‘While participants anticipated that injectable PrEP would improve HIV prevention outcomes by reducing adherence burden and HIV-related stigma...’ | **p. 2, lines 28–29** |
| **Comment R2.2 Reviewer 2**  **Original text: ...however, they were hopeful, dependent on the demonstrated success of injectable PrEP.**  “dependent on the demonstrated success of injectable PrEP.” Unclear what that means. | Revised to specify what ‘demonstrated success’ means in practice.  REVISED TEXT (p. 2, lines 41–42): ‘...however, their support for injectable PrEP was contingent on witnessing its real-world effectiveness ,specifically, safe administration, manageable side effects, and acceptable outcomes, in their own campus clinic settings before full endorsement.’ | **p. 2, lines 31–33** |
| **Comment R2.3 Reviewer 2**  **Original text: ...young people remain disproportionately affected despite extensive national prevention programmes [1, 2].**  Examples of national prevention programmes would be helpful. | Thank you. Specific programme names have been added.  REVISED TEXT (p. 2, lines 56–57): ‘...despite extensive national prevention programmes, including the HIV Counselling and Testing (HCT) Campaign, the Determined, Resilient, Empowered, AIDS-free, Mentored and Safe (DREAMS) initiative, and the national oral PrEP programme [1, 2].’ | **p. 2, lines 45–48** |
| **Comment R2.4 Reviewer 2**  **Original text: KwaZulu-Natal consistently records the highest HIV prevalence nationally, underscoring the urgent need for differentiated, youth-centered prevention approaches [3].**  Are there certain contextual factors in KZN that contribute to the high HIV prevalence? Are young people disproportionately burdened by HIV in KZN similar to nationally? | Thank you. We have expanded this sentence to describe key KZN-specific contextual drivers and the disproportionate burden on young women.  REVISED TEXT (p. 2, lines 57–59): ‘KwaZulu-Natal consistently records the highest HIV prevalence nationally, driven by intersecting factors including gender inequality, high poverty rates, migrant labour patterns, and elevated rates of gender-based violence. Young women aged 15–24 in KZN bear a disproportionate burden, with HIV incidence rates among the highest globally, underscoring the urgent need for differentiated, youth-centred prevention approaches [3].’ | **p. 2–3, lines 48–53** |
| **Comment R2.5 Reviewer 2**  **Original text: ...lifestyle patterns [5, 6].**  What does it mean for a young person to struggle with daily pill-taking due to lifestyle patterns? | Thank you. ‘Lifestyle patterns’ has been expanded to specify the relevant behaviours and circumstances. By “struggle with daily pill-taking due to lifestyle patterns,” we refer to common characteristics of young people’s daily routines that can make consistent medication adherence challenging.  REVISED TEXT (p. 2, lines 64–65): ‘...lifestyle patterns such as irregular routines, frequent travel between home and campus, and socially dynamic environments that make discreet, consistent daily pill-taking difficult to sustain [5, 6]. Long-acting injectable PrEP may offer an important alternative, as it reduces the need for daily adherence and may better align with the realities of young people’s lives.’ | **p. 3, lines 57–60** |
| **Comment R2.6 Reviewer 2**  While it is noted that South Africa has permitted a phased rollout of injectable, please specify to what extent injectable PrEP has been rolled out in KZN and at university clinics. | Thank you. Rollout status clarified in both the Background (via R1.1 revision) and the Study Setting section, specifying that no campus health clinic in KZN had yet introduced injectable PrEP at the time of study.  ADDED TEXT (p. 5, after line 137): ‘At the time of this study, injectable PrEP had not been introduced at any campus health clinic in KwaZulu-Natal, as national implementation planning, including finalisation of clinical guidelines and training protocols, was still underway.’ | **p. 5, lines 119–129** |
| **Comment R2.7 Reviewer 2**  **Original text: Each clinic is staffed by multidisciplinary teams comprising PHC nurses, part-time medical doctors, HIV counsellors, and health promoters.**  Different cadres of PHC – who does what related to PrEP? | A paragraph describing each cadre’s HIV prevention role has been added to the Study Setting section.  ADDED TEXT (p. 5, after rollout clarification): ‘PHC nurses conduct HIV risk assessments, provide PrEP counselling, initiate and manage oral PrEP, and administer injectable medications, including contraceptives. Medical doctors provide clinical oversight, manage complex cases, and authorise prescriptions. HIV counsellors conduct pre- and post-test counselling, adherence support, and facilitate informed decision-making. Under current NIMART and PrEP guidelines, professional nurses may initiate and prescribe oral PrEP independently.’ | **p. 5–6, lines 123–129 (Study Setting)** |
| **Comment R2.8 Reviewer 2**  **Original text: We employed purposive sampling to recruit PHC workers with relevant knowledge and experience in HIV prevention service delivery [22].**  Please explain what is meant by “PHC workers with relevant knowledge and experience in HIV prevention service delivery.” Eligibility criteria part c indicates that PHC were eligible if they were directly involved in HIV prevention services. How was a PHC worker determined to have the “relevant knowledge and experience”? | Thank you, the phrase has been replaced with precise, operationalisable language tied to the eligibility criteria.  REVISED TEXT (p. 5, lines 141–142): ‘We employed purposive sampling to recruit PHC workers directly involved in HIV prevention service delivery as part of their defined job role [22].’ PHC workers were considered to have relevant knowledge and experience if they were directly engaged in services such as HIV testing and counselling, provision of oral PrEP, condom distribution, sexual and reproductive health counselling, or other HIV prevention-related activities. | **p. 6, lines 132–133** |
| **Comment R2.9 Reviewer 2**  **Original text: ...by the first author (NNM), a female Master’s student trained in qualitative research methods.**  I do not think it's relevant to specify the gender of the first author. | Thank you, The word ‘female’ has been removed.  REVISED TEXT (p. 5, line 168): ‘...by the first author (NNM), a Master’s student trained in qualitative research methods.’ | **p. 7, line 151** |
| **Comment R2.10 Reviewer 2**  **Original text: All healthcare workers approached agreed to participate in the study; there were no refusals or non-responses.**  What is meant by “all healthcare workers approached agreed to participate in the study” as it appears from earlier narrative that the researcher did not individually recruit participants – rather participants opted in to contacting researchers if they were interested in participating based on the flyer. | Thank you. The statement has been corrected to accurately reflect the opt-in recruitment process.  REVISED TEXT (p. 6, lines 172–174): ‘All eligible staff members who contacted the researcher in response to the study flyer agreed to participate; there were no withdrawals of consent or non-completions following initial expression of interest.’ | **p. 7, lines 155–160** |
| **Comment R2.11 Reviewer 2**  **Original text: ...piloted with three PHC workers from non-study clinics to refine question clarity and flow.**  Was the guide piloted at non-study university health clinics? | Thank you. Clarified that piloting was conducted at non-study campus health clinics at the same university, and that pilot data were excluded from analysis.  REVISED TEXT (p. 6, lines 177–178): ‘...piloted with three PHC workers from non-study campus health clinics at the same university to refine question clarity and flow. Pilot data were not included in the analysis.’ | **p. 7, lines 162–164** |
| **Comment R2.12 Reviewer 2**  **Original text: Audio recordings were transcribed verbatim by the first author, with transcripts verified for accuracy by the co-authors.**  Did both co-authors verify accuracy of transcripts? | Thank you. We have clarified that both co-authors verified accuracy independently.  REVISED TEXT (p. 6, lines 190–191): ‘...with transcripts verified for accuracy by both co-authors independently (DG; NM).’ | **p. 7, lines 171–172** |
| **Comment R2.13 Reviewer 2**  **Original text: We employed Babchuk’s 10-step thematic analysis process [25], which combines systematic rigor with interpretive flexibility.**  please describe how Babchuk's analysis process was utilized in this work. | Thank you, All 10 steps have been described explicitly as applied in this study.  REVISED TEXT (p. 6, lines 192–194): ‘We employed Babchuk’s 10-step thematic analysis process [25], applied as follows: (1) reading all transcripts to gain overall familiarity; (2) applying open codes to discrete data segments; (3) grouping similar codes into categories; (4) developing a preliminary codebook; (5) testing and refining codes through re-reading; (6) organising categories into higher-order themes; (7) defining and naming themes; (8) writing analytic memos; (9) reviewing themes against all data; and (10) producing the final thematic structure.’ | **p. 7–8, lines 173–179** |
| **Comment R2.14 Reviewer 2**  **Original text: Trustworthiness was established using Lincoln and Guba’s criteria [26].**  It would be helpful to define Lincoln and Guba's 4 criteria before describing how these were achieved in this work. | Definitions of all four criteria have been added before the descriptions of how each was achieved.  REVISED TEXT (p. 7, line 204): ‘Trustworthiness was established using Lincoln and Guba’s four criteria: credibility (internal validity of findings), dependability (consistency and auditability of the research process), confirmability (freedom from researcher bias), and transferability (applicability to other contexts) [26].’ | **p. 8, lines 190–193** |
| **Comment R2.15 Reviewer 2**  **Original text: A detailed codebook was developed iteratively, with codes defined, refined, and organized hierarchically.**  More is needed to understand who conducted the data analysis – how many people were involved in the data analysis? How many people analyzed each transcript? Who developed the codebook? What was the role of the supervisor (Is the supervisor a co-author? If so, identify by initials.) | Thank you, A paragraph explicitly describing authorial roles in analysis has been added.  ADDED TEXT (p. 6, after line 200): ‘The primary analysis was conducted by the first author (NNM), who coded all 18 transcripts. A preliminary codebook was developed inductively by NNM and then reviewed and refined in discussion with both supervisors (DG; NM), who are co-authors. DG and NM independently reviewed a subset of five transcripts to verify coding consistency; discrepancies were resolved through discussion until consensus was reached. The final thematic structure was reviewed and approved by all three authors.’ | **p. 8, lines 181–188 (Data Analysis)** |
| **Comment R2.16 Reviewer 2**  **Original text: Transferability was supported by a thick description of the study context...**  Recommend rephrasing to “comprehensive description of study context….” | Thank you. ‘Thick description’ replaced with ‘comprehensive description’ as recommended.  REVISED TEXT (p. 7, lines 213–214): ‘Transferability was supported by a comprehensive description of the study context, setting, participants, and procedures, enabling readers to assess applicability to other contexts.’ | **p. 9, lines 200–202** |
| **Comment R2.17 Reviewer 2**  **Original text: Authenticity was ensured through fair representation of diverse perspectives, including minority views, and attention to power dynamics, ensuring marginalized voices were acknowledged.**  Recommend rephrasing the authenticity statement. | Thank you, Adopted the suggested phrasing; ‘marginalized’ corrected to ‘marginalised’ for South African English consistency.  REVISED TEXT (p. 7, line 218): ‘Authenticity was ensured through fair representation of diverse perspectives, including minority views, and attention to power dynamics, ensuring marginalised voices were acknowledged.’ | **p. 9, line 204** |
| **Comment R2.18 Reviewer 2**  **Original text: ...predominantly female (88.9%) and mid-to-late career (mean age 52.4 years, SD 13.8; mean clinical experience 16.6 years, SD 11.2).**  Separate out age and clinical experience as age is unrelated to career duration. | Thank you, Age and clinical experience now reported separately; ‘mid-to-late career’ removed.  REVISED TEXT (p. 7, lines 231–234): ‘The 18 participants comprised 10 PHC nurses (55.6%), 3 medical doctors (16.7%), and 5 HIV counsellors (27.8%), predominantly female (88.9%). Participants had a mean age of 52.4 years (SD 13.8) and a mean of 16.6 years of clinical experience (SD 11.2), reflecting distinct dimensions of professional background.’ | **p. 9, lines 213–216** |
| **Comment R2.19 Reviewer 2**  Please explain the role of these nurses, MDs, and HIV counsellors in HIV service delivery. Can nurses prescribe HIV prevention medication? Who can administer injections? Who conducts HIV tests? | Thank you, addressed together with R2.7. The Study Setting section now includes a paragraph for each cadre describing its role, prescribing authority, and scope in HIV prevention.  Please see action under R2.7. | **p. 5–6, lines 123–129 (Study Setting)** |
| **Comment R2.20 Reviewer 2**  **Original text: ...while also acknowledging their doubts associated with the nature of the target population for injectable PrEP.**  Unclear what is meant by “acknowledging their doubts associated with the nature of the target population for injectable PrEP.” The corresponding quote seems to express concern about side effects and health outcomes. | Thank you. The unclear phrase has been replaced with language that accurately reflects participants' expressions.  REVISED TEXT (p. 14, lines 438–440): ‘...while also acknowledging their concerns about real-world effectiveness, potential unanticipated side effects, and uncertainty about how the intervention would perform in campus health settings.’ | **p. 16, lines 345–348** |
| **Comment R2.21 Reviewer 2**  **Original text: P12 Medical Doctor quote in Theme 1.**  P.9 quote beginning at line 199: seems better suited for Theme 4. | Thank you. The knowledge component of the P12 quote (cabotegravir, efficacy, dosing) justifies its retention in Theme 1. We have retained it and added a cross-reference sentence directing readers to Theme 4, where the conditional optimism is developed fully.  ADDED TEXT (p. 10, after line 318): ‘The conditional optimism expressed in this quote, knowledge paired with a call for local evidence before full endorsement ,reflects a broader stance explored in depth in Theme 4.’ | **p. 12, lines 250–252** |
| **Comment R2.22 Reviewer 2**  **Original text: ...information most often obtained through online news and peer networks.**  It is noted that information is most often obtained through online news and peer networks. Please if possible describe further what “online news” means, and additionally, what other sources of information participants identified. | Thank you. Sources have been specified to distinguish between healthcare worker cadres.  REVISED TEXT (p. 11, lines 344–349): ‘...information was most often obtained through informal channels, including online news sources (such as health news websites and social media platforms), peer conversations with colleagues, and, among doctors, international research publications. Notably, none reported receiving formal training or structured information through official health system channels. This reliance on informal and fragmented information sources directly contributed to the clinical protocol confusion described in Theme 2.’ | **p. 12–13, lines 271–281** |
| **Comment R2.23 Reviewer 2**  This finding (information sources) seems more related to theme 2. | Thank you. We have retained the finding in Theme 1 as it concludes the explanation of variable awareness, but added an explicit bridging sentence linking to Theme 2 (included in the R2.22 action above).  Please see action under R2.22. | **p. 12–13, lines 271–281** |
| **Comment R2.24 Reviewer 2**  **Original text: As a result, participants’ limited understanding of eligibility guidelines created uncertainty and hesitation...**  Please provide a quote to support the finding that limited understanding of eligibility guidelines created uncertainty and hesitation. | Thank you. The P18 HIV Counsellor quote already present immediately precedes this summary statement. We have ensured the sequence is explicit.  Confirmed: P18 quote (‘Without clear guidelines, it’s hard to counsel effectively.’) immediately precedes the summary at p. 12, lines 390–393. No additional change required. | **p. 14, lines 309–312** |
| **Comment R2.25 Reviewer 2**  **Original text: Participants felt that such a choice could empower users, allowing them to select a prevention method that best fits their personal preferences and lifestyles.**  It is not evident through the provided quotes how choice could empower users. | Thank you. Text revised to ground the agency/choice framing in participants’ actual words, the contraceptive choice analogy, rather than using ‘empower’ as an unsupported interpretive claim.  REVISED TEXT (p. 13, lines 413–418): ‘This optimism was grounded in the perception that injectable PrEP offers individuals greater agency over their prevention decisions. Participants drew explicit parallels to contraceptive method choice, noting that having options allowed clients to select what aligned with their circumstances, thereby reducing reliance on a single modality that might not suit everyone.’ | **p. 15, lines 326–330** |
| **Comment R2.26 Reviewer 2**  **Original text: Theme 4 contained only P12 and P11 (both Medical Doctors).**  Both quotes provided are from MDs. Are there supporting quotes from nurses or counsellors? | Thank you. Quotes from a PHC Nurse (P03) and an HIV Counsellor (P14) have been added to Theme 4, along with a bridging sentence emphasing the cross-cadre nature of this theme.  ADDED TEXT (p. 14, after line 463): Bridging sentence plus P03 PHC Nurse quote and P14 HIV Counsellor quote, demonstrating that conditional optimism was expressed across all three cadres. | **p. 17, lines 366–383 (Theme 4)** |
| **Comment R2.27 Reviewer 2**  In Theme 1 findings, authors nicely describe how findings varied across cadres; however in findings for themes 2–4, this specificity is missing. While obviously these findings are not generalizable throughout each cadre, it would be helpful to identify if there were any differences/similarities across cadres. | Thank you. Cadre-level narrative has been added to Themes 2, 3, and 4. Theme 4 now includes quotes from all three cadres (see R2.26). Where data did not yield sufficiently distinct quotes for Themes 2 and 3, this is acknowledged in the narrative.  Please see R2.26 for Theme 4 additions. Cross-cadre narrative added within Themes 2 and 3, where supported by data. | **p. 11–17, lines 228–383 (Themes 2–4)** |
| **Comment R2.28 Reviewer 2**  Generally, recommend providing more direct quotes , perhaps at least one per cadre, to support findings. The quotes are very rich! | Thank you, all themes reviewed: Theme 1 now has quotes from MD (P12), PHC Nurse (P05), HIV Counsellor (P16). Theme 2: PHC Nurse (P07), HIV Counsellor (P18). Theme 3: HIV Counsellor (P15), PHC Nurse (P02). Theme 4 now includes all three cadres (see R2.26).  Please see R2.26 for new quotes added to Theme 4. | **p. 11–17, lines 228–383 (all Themes)** |
| **Comment R2.29 Reviewer 2**  **Original text: These findings reveal that PHC workers occupy a position of informed hesitancy, they recognize injectable PrEP’s potential benefits...**  Recommend rephrasing to, “These findings reveal that PHC workers occupy a position of informed hesitancy, in that they recognize injectable PrEP's potential benefits…”. | Thank you. The suggested phrasing has been adopted with minor grammatical correction (‘recognise’ for South African English).  REVISED TEXT (p. 15, lines 495–496): ‘These findings reveal that PHC workers occupy a position of informed hesitancy, in that they recognise injectable PrEP’s potential benefits while expressing legitimate concerns about their preparedness to implement it safely and effectively.’ | **p. 18, lines 405–407** |
| **Comment R2.30 Reviewer 2**  **Original text: Beyond awareness and knowledge levels, PHC workers expressed diverse perceptions about injectable PrEP...**  Did PHC workers express diverse perceptions? The findings as presented seem to overwhelming suggest that providers were interested, optimistic, yet cautious about injectable PrEP. From the evidence presented (Wheelock, Rubincam, etc) – are these specific to injectable PrEP? Who are the participants in each study (healthcare workers?)? | Thank you, Revised to characterise perceptions as cautious optimism varying by cadre (not broadly ‘diverse’); explicitly stated that cited studies concern oral PrEP; named participant populations of each study.  REVISED TEXT (p. 16, lines 502–521): Paragraph now opens ‘PHC workers expressed nuanced perceptions… cautious optimism rather than sharply divergent views, the texture and basis of that optimism varied meaningfully across cadres.’ Wheelock et al. identified policymakers/HCWs/NGO representatives across seven countries; Rubincam et al. identified key populations in South Africa; Kambutse et al. identified HCWs and community members in Rwanda. | **p. 19, lines 410–423** |
| **Comment R2.31 Reviewer 2**  **Original text: Professional backgrounds significantly mediate understanding and acceptance of new HIV prevention methods.**  What is meant by “professional backgrounds significantly mediate understanding and acceptance of new HIV prevention methods”? Is it the cadre of PHC worker? The amount of experience in HIV prevention service delivery? Unclear how that statement connects to the findings presented here. | Thank you. The vague statement has been replaced with specific language identifying cadre as the operative dimension of professional background.  REVISED TEXT (p. 16, lines 519–521): ‘The variations observed among nurses, doctors, and HIV counsellors reflect professional background, specifically cadre, with its associated differences in training pathways, scope of practice, and access to medical literature, as a key mediating factor; in this study, doctors demonstrated more detailed knowledge than nurses or counsellors.’ | **p. 26, lines 604–610** |
| **Comment R2.32 Reviewer 2**  **Original text: Similarly, Pleaner, et al [31] observed that many providers expressed concerns about possible unintended consequences, with many worried that PrEP might encourage risky sexual behaviour. This explains why their perceptions varied considerably...**  Did this work have similar findings to Pleaner (unintended consequences, encourage risky sexual behaviour)? Seems like findings are more in line with Asabor. | Thank you. Moral hazard concerns were not prominent in our study. Explicit distancing text added, aligning our findings with Asabor et al.  ADDED TEXT (p. 17, after line 539): ‘Notably, such concerns about moral hazard were not prominent in the present study; findings align more closely with Asabor et al.’s structural-barriers framing and cautious optimism, with participants’ reservations centring on implementation readiness, training gaps, and clinical uncertainty rather than risk compensation.’ | **p. 20, lines 439–442** |
| **Comment R2.33 Reviewer 2**  **Original text: Multiple studies across African contexts reveal persistent obstacles.**  Again, is the evidence in this paragraph specific to injectable PrEP? | Thank you. A framing sentence has been added noting these studies concern oral PrEP contexts, and each study’s population is now named in-text.  REVISED TEXT (p. 18, lines 567–575): ‘Although the following evidence derives primarily from oral PrEP contexts, the underlying adherence dynamics are equally applicable to injectable PrEP implementation in campus settings, as the structural and behavioural barriers driving non-adherence are not formulation-specific. While direct evidence from university student populations remains limited, studies among comparable young populations across African contexts reveal persistent obstacles that are likely to resonate in campus settings.’ Each cited study’s population is now named. | **p. 21, lines 472–487** |
| **Comment R2.34 Reviewer 2**  **Original text: Healthcare workers consistently recognize that medication adherence is a complex, multilevel challenge...**  The evidence discussed here – is it specific to students? How is it relevant to the presented work? | Thank you. A framing sentence has been added acknowledging these studies are from oral PrEP contexts and explaining their relevance to the campus setting.  ADDED framing sentence (p. 20, before line 647): ‘The participants in this study recognised, through their daily clinical experiences with oral PrEP, that medication adherence is a complex, multilevel challenge that extends far beyond individual motivation, and this understanding underpinned their optimism about injectable PrEP as an adherence solution. Although the following evidence derives primarily from oral PrEP contexts, the underlying adherence dynamics are equally applicable to injectable PrEP implementation in campus settings.’ | **p. 24, lines 539–557** |
| **Comment R2.35 Reviewer 2**  Paragraph beginning at p. 22 line 499 is a repeated paragraph (p. 17 line 374). | We thank the reviewer for identifying this. The duplicate paragraph has been deleted.  Duplicate paragraph removed. | **p. 17, duplicate paragraph** |
| **Comment R2.36 Reviewer 2**  While evidence from other studies is brought into the discussion, it is not always clear how this evidence does or does not support or align with the current work. At times, the discussion seems more like a review of the literature, and less contextualizing the work within the current evidence base. Additionally, it is not clear if the evidence presented is relevant to the current work. Connect findings to practical guidance for implementation. Discuss how findings can inform future introduction of HIV prevention technologies (long-acting lenacapavir). | Thank you. This overarching concern guided our entire Discussion revision: (1) Explicit linking language after each cited study. (2) New ‘Implementation Considerations for Campus Settings’ subsection. (3) Discussion of how ‘informed hesitancy’ and cadre-specific gaps can guide pre-implementation training for lenacapavir. (4) Client-level barriers cited only to explain HCW optimism.  The revised Discussion addresses this overarching concern through four targeted actions: (1) explicit linking language has been added after each cited study to connect external evidence directly to our campus clinic findings; (2) a new ‘Implementation Considerations for Campus Settings’ subsection has been inserted, discussing academic calendar risks (missed injections during long university holidays and the associated pharmacokinetic tail risk of seroconversion and ARV resistance), workload demands on campus clinic staff (cold-chain storage, intensified counselling, and additional appointment load), and mitigation strategies including bridging oral PrEP, aligned scheduling, and re-testing protocols; (3) the concept of ‘informed hesitancy’ and cadre-specific knowledge gaps are now explicitly linked to practical guidance for pre-implementation training ahead of the lenacapavir rollout; and (4) client-level adherence barriers are cited only to explain and contextualise HCW optimism about injectable PrEP, rather than as standalone literature review content. Individual revisions addressing specific evidence-to-findings links are detailed under R2.30–R2.34. | **p. 18–29, lines 385–681 (Discussion)** |
| **Comment R2.37 Reviewer 2**  **Original text: The limited awareness among nurses is particularly significant...**  Earlier under Theme 1 it is noted ‘Among the ten nurses interviewed, seven reported having some knowledge of injectable PrEP, whereas three indicated limited or no awareness of it.’ This contradicts line 529. | Thank you, The contradicting statement has been corrected to align with Theme 1 data.  REVISED TEXT (p. 23, lines 725–726): ‘The variable awareness among nurses is particularly significant given their central role in primary healthcare service delivery in South Africa. While seven of ten nurses demonstrated some knowledge of injectable PrEP, three had limited or no awareness, indicating a significant proportion who would require foundational knowledge-building before implementation.’ | **p. 27, lines 612–616** |
| **Comment R2.38 Reviewer 2**  **Original text: The uncertainty surrounding eligibility criteria suggests that PHC workers have not yet begun implementing injectable PrEP.**  Previously, the authors note that injectable PrEP implementation has not yet begun, line 562 seems to say that this is due to uncertainty surrounding eligibility criteria. Is that factual? Or has it not begun for other reasons (not yet rolled out in South Africa)? This is not clear. | Thank you, The misleading causal implication has been corrected. Implementation has not begun due to national planning processes, not eligibility uncertainty.  REVISED TEXT (p. 24, lines 763–764): ‘The uncertainty surrounding eligibility criteria among PHC workers reflects the absence of formal pre-rollout training rather than any structural barrier to national implementation. Injectable PrEP has not yet been introduced in South African campus clinics because national implementation planning, including finalisation of clinical guidelines and training protocols, was still underway at the time of this study.’ | **p. 28, lines 648–652** |

We hope the revised manuscript and this detailed response satisfactorily address all reviewer concerns. We remain available to provide any further clarification if needed.

Sincerely,

**Nomvuselelo Nomzamo Mbatha, Nomakhosi Mpofana, and Dumile Gumede**
